# The Role of Fas-FasL Signaling Pathway in Induction of Apoptosis in Patients with Sulfur Mustard-Induced Chronic Bronchiolitis

**DOI:** 10.1155/2010/373612

**Published:** 2011-01-13

**Authors:** Gila Pirzad, Mahvash Jafari, Sasan Tavana, Homayoon Sadrayee, Saeid Ghavami, Arezoo Shajiei, Mostafa Ghanei

**Affiliations:** ^1^Chemical Injury Research Center, Faculty of Medicine, Baqiyatallah University of Medical Sciences, Tehran 1956837173, Iran; ^2^Department of Biochemistry, Faculty of Medicine, Baqiyatallah University of Medical Sciences, Tehran 1956837173, Iran; ^3^Clinical Research and Development Center, Shahid Modarres Hospital, Shahid Beheshti University, Tehran 1991733981, Iran; ^4^Department of Anatomy, Faculty of Medicine, Baqiyatallah University of Medical Sciences, Tehran 14359151371, Iran; ^5^National Training Program in Allergy and Asthma, University of Manitoba, Winnipeg, MB, Canada R3T 2N2; ^6^Biotechnology Center, Faculty of Allied Medicine, Iran University of Medical Sciences, Tehran 1956837173, Iran

## Abstract

Sulfur mustard (SM) is an alkylating agent that induces apoptosis and necrosis in cells. Fas-Fas ligand (FasL) interaction could induce apoptosis as well. In this study, it was hypothesized that apoptosis might play an important role in the pathogenesis of SM-induced lung injury via Fas-FasL signaling pathway. In a case-control study, Fas and FasL levels, caspase-3 activity and percent of apoptotic cells were measured in bronchoalveolar lavage (BAL) fluid of patients 20 years after exposure to sulfur mustard and compared with the control group. Results show that Fas and FasL levels were significantly higher in BAL fluid cells in patients group compared with the control (*P* = .001). No significant differences were observed between mild and moderate-severe groups. BAL fluid cells caspase-3 activity was not significantly different among the mild, moderate-severe, and control groups. The data suggest that Fas-FasL-induced apoptosis was impaired in BAL fluid cells of SM-exposed patients which might be one of the initiators of pathogenesis in SM-induced lung injury in these patients.

## 1. Introduction

Sulfur mustard (SM) is a vesicant compound warfare agent that causes acute and chronic effects on different organs following exposure. It was used against Iranians by Iraqies during Iraq-Iran ware. The eyes, the skin, and the respiratory tract are three principal target organs of SM toxicity [1, 2]. A main late pulmonary complication of SM is bronchiolitis obliterans (BOs) [3–5]. However, the mechanism of SM-induced respiratory injuries is not fully understood.

SM is an alkylating agent causing single- and double-strand breaks in the DNA and also reacts with RNA, proteins, and lipid membranes. Thus, it leads to a disordered cell metabolism, causing cell death [6, 7]. In vitro and in vivo studies showed that SM induces time- and dose-dependent apoptosis (physiological cell death) and necrosis (pathological cell death) in cells [8–11].

Two major pathways have been described to trigger apoptosis, namely the extrinsic pathway (death receptor pathway) and the intrinsic pathway (mitochondrial pathway) within the cell. Interestingly, both pathways seem to be involved in SM-induced apoptosis [6, 12]. The extrinsic pathway is activated by ligand-activated death receptors such as Fas ligand- (FasL-) Fas [13]. The binding of Fas-FasL activates caspases, cysteine proteases that recognize aspartate at their substrate cleavage site, and induced apoptosis [14]. SM may develop susceptibility to mutations in tumor suppressor, such as p53, to reduce bcl-2, and to activate caspase-3 in vitro [15].

SM injury to the respiratory system has been related to apoptotic cell death. Several investigators have shown that SM induces apoptosis in lung-derived cells and that the effector caspase-3 is activated in a dose- and time-dependent manner after SM injury [12, 15]. In vivo study with rodent pulmonary tissue exposed to SM showed increased gene expression of apoptosis-related genes [16]. However, little is known about the signal transduction pathways activated by long-term effects of SM. The purpose of the present study was to investigate the mechanism of cell death via Fas-FasL pathway that occurred in brochoalveolar lavage (BAL) fluid of patients 20 years after exposure to sulfur mustard. Understanding the molecular and cellular pathways activated in response to SM exposure can lead to therapeutic strategies for prevention or treatment of SM toxicity.

## 2. Materials and Methods

### 2.1. Patients Group

Twenty patients with history of exposure to a single high dose of SM from 1985 to 1987 during the Iran-Iraq war who suffered from persistent respiratory and chest discomfort, shortness of breath, cough, and exercise intolerance were reviewed systematically. These patients were selected among all those who were referred to the Emergency Department of Baqiyatallah Hospital as the main referral center for chemically injured patients in Tehran, Iran. The documentation of SM exposure was based on official certification issued by the Iranian Veterans Foundation, which is the official center for compensation of war-disabled victims. Patients with a history of smoking and occupational exposure to toxic agents and having dusty jobs were excluded from the study.

### 2.2. Control Group

Six healthy volunteers, nonsmoking individuals with no history of SM exposure and no signs or symptoms of respiratory disease were included as the control group. Ethical approval for this research was obtained from the Ethics Committee of the Baqiyatallah University of Medical Sciences, and informed consent was obtained from all patients.

### 2.3. Pulmonary Function Test (PFT)

To assess pulmonary function using spirometry (Hi801 Chest M.I. Spirometer), the residual volume (RV), forced vital capacity (FVC), forced expiratory volume in 1 second (FEV1), and FEV1/ FVC were measured. Based on postbronchodilator FEV1, patients were divided into two groups: mild (*n* = 10) and moderate-to-severe (*n* = 10) pulmonary dysfunction [17].

### 2.4. Bronchoscopy and BAL Sampling

BAL was performed in all subjects using a flexible fiber-optic bronchoscope (Olympus BF1T, Tokyo, Japan). The upper respiratory tract was anesthetized with 2% lidocaine. Atropine (0.75 mg intramuscularly) was administered before the procedure. Supplemental oxygen was given throughout the procedure, and the oxygen saturation was monitored by continuous pulse oxymeter. The bronchoscope was wedged for lavage in the middle lobe segmental bronchus, and four 60-mL aliquots of sterile physiological saline solution warmed to 37°C were infused. The fluid was immediately recovered by gentle suction after each instillation. After that brushing was obtained from each patient via disposable cytology brush (Olympus BC-202D-C10, Tokyo, Japan) in order to increase epithelial cell number. The cell suspension has been centrifuged at 500 g for 10 minutes in 4°C. Cell plates were washed by cold PBS buffer and were stored in −70°C. For cell sampling, a catheter was introduced via the sampling channel. The sleeve was retracted, and the bristles were exposed. The brush was then rubbed against the epithelial surface under direct visual guidance. The brush was then retracted, and the dissociated cells were well recovered by vortexing the brush in BAL for several seconds; brushing was repeated in three to four locations in the distal trachea and proximal main stem bronchi [18].

### 2.5. Fas Level Assay

BAL fluid cells Fas level was measured using the RayBio human Fas ELISA kit (Cat NO. ELH-Fas-001, USA). Standards and samples were pipetted into the wells. Presented Fas in samples was bound to the wells by the immobilized antibody. The wells were washed, and biotinylated antihuman Fas antibody was added. After washing away unbound biotinylated antibody, HRP-conjugated streptavidin was pipetted to the wells. The wells were again washed, and a tetramethylbenzidine (TMB) substrate solution was added to the wells and color developed in proportion to the amount of Fas bound. The stop solution has changed the color from blue to yellow, and the intensity of the color was measured at 450 nm.

### 2.6. Fas Ligand Level Assay

BAL fluid cells FasL level was measured with Fas Ligand “sandwich” ELISA kit (Cat no. QIA27, Oncogene Research Products, USA). Samples and biotinylated detector monoclonal antibody were pipetted into the wells and allowed to incubate for three hours. During this time, presented FasL was bound to the capture and detecting antibodies. Unbound material was washed away, and horseradish peroxidase-conjugated streptavidin was added, which is bound to the detector antibody. The horseradish peroxidase catalyzed the conversion of the chromogenic substrate tetramethylbenzidine (TMB) from a colorless solution to a blue solution (or yellow after the addition of stop reagent). Its intensity was proportional to the amount of FasL protein in the sample. The colored reaction product was quantified using a spectrophotometer by the construction of a standard curve using known concentrations of FasL (provided lyophilized). The concentration of FasL in the sample could be determined by comparing the absorbance obtained from a sample containing an unknown amount of FasL with that standard.

### 2.7. Caspase-3 Activity Assay

Caspase-3 activity was measured with caspase ELISA kit (Cat no. C2087-12, CPP32, Apotain, Yama, SCA-1, US Biological Company). Briefly, 100 *μ*L cell lysate or 1 unit recombinant human caspase-3 (positive control) was added to antibody-coated well. The plate was tightly covered and incubated at 37°C for 1 hour. Then, the solutions were removed and after washing 3 times with 150 *μ*L incubation buffer. Then, 94 *μ*L incubation buffers, 5 uL DEVD-AFC, and 1 *μ*L DTT were added to each well. The plate was tightly covered and incubated at 37°C for 2–4 hours. The samples were read at Ex: 370–425 nm and Em: 490–525 nm in a fluorescence microtiter plate reader. The notable increase in caspase-3 activity was determined by comparing the results with uninduced control.

### 2.8. Detection of Apoptosis Using Annexin V

To detect phosphatidylserine, annexin V-FITC (Trevigen Inc., Gaithersburg, USA or Boehringer Mannheim, GmbH, Germany) was used in a combination with propidium iodide (PI) using Apopnexin FITC Apoptosis Detection Kit (Katayama Chemical, Osaka, Japan). The FITC-Annexin V fluorescence was read with the FL1 photomultiplier tube, and PI fluorescence was detected using the FL3 channel.

### 2.9. Protein Concentration Assay

The total protein concentration in BALF was measured by Branford's method31 using bovine serum albumin as standard [19].

### 2.10. Statistical Analysis

SPSS 15.0 (SPSS Inc, Chicago, IL, USA) was used for statistical analysis. Comparisons between the patients and control groups and also between subgroups of patients were assessed by one-way ANOVA. Significant differences between means were analysed by the post-hoc test using Scheffe's F. Spearman's rank correlation coefficient was used to analyse correlations between serum markers. Data was shown as means ± SD. *P*-values less than .05 were considered statistically significant.

## 3. Results

### 3.1. Control and Case Group Demographic Information

Comparison of mean age among the control (38.7 ± 6.1) and patients groups with mild (39.5 ± 5.4) and moderate-to-severe (43.5 ± 5.3) disease did not show significantly different (*P* = .24) (Table 1). 

### 3.2. PFT Finding

As described in Table 2, FVC, FEV1 and FEV1/FVC ratio was significantly lower in patients with moderate-to-severe and FVC in patients with mild respiratory disease compared with the control group. FEV1/FVC ratio was significantly higher in patients with mild compared with moderate-to-severe respiratory disease (*P* = .001).

### 3.3. Differential Cell Count

Differential BAL fluid cell count showed that there was not significant difference in polymorphonuclear (PMN), macrophages, and lymphocytes between the severe and the control group. There was only a difference observed in PMN between the mild and control group. PMN was significantly lower in mild group (*P* < .05) (Table 3). 

### 3.4. BAL Fluid Fas and FasL Levels in the Patient and Control Group

**Figure 1:**
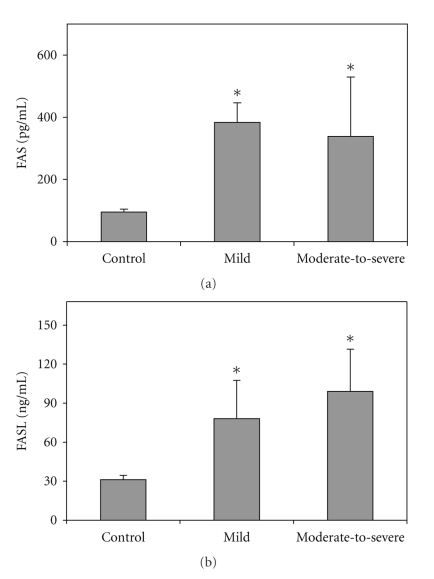
Fas and FasL levels in the normal, mild and moderate-to-severe patient groups. Values are shown as mean ± SD. *P < .05 versus control.

As displayed in Figure 1, the Fas and FasL levels in the BAL fluid cells of patient with mild and moderate-to-severe lung dysfunction are significantly higher than the control group. There were not any significant differences between the mild and moderate-to-severe groups (*P* > .05).

### 3.5. BAL Fluid Caspase-3 Activity in the Patient and Control Group

**Figure 2:**
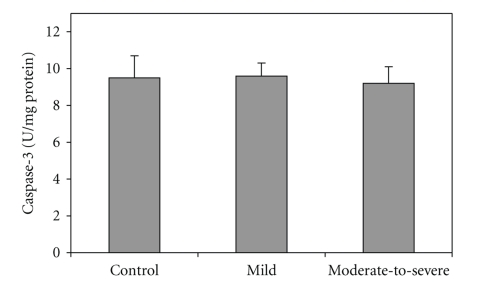
Caspase-3 activity in the normal, mild, and moderate-to-severe patient groups. Values are shown as mean ± SD.

BAL fluid cells Caspase-3 activity in the patient and control group is shown in Figure 2. There was not any significant difference in BAL fluid cells caspase-3 activity between the control and patient groups (*P* > .05).

### 3.6. Percent of Apoptotic and Necrotic Cells in the Patient and Control Group

**Figure 3:**
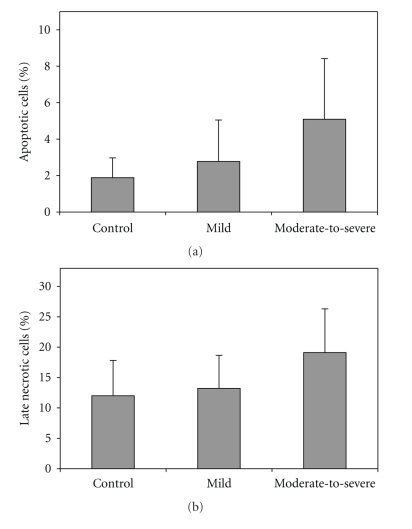
Percent of apoptotic and late necrotic cells of BAL fluid cells in the normal, mild, and moderate-to-severe patient groups. Values are shown as mean ± SD.

As seen in Figure 3, there was not any significant difference in percent of apoptotic and necrotic cells of BAL fluid between the control and patient groups. Percent of apoptotic cells is lower than late necrotic cells.

## 4. Discussion

Apoptosis, programmed cell death, is known to participate in various biological processes such as development, maintenance of tissue homeostasis, and elimination of cancer cells. Malfunctions of apoptosis have been implicated in many forms of human diseases [20, 21]. The potential role of the Fas receptor pathway in asthma has been intensively studied during recent years, albeit with contradictory results. Some studies have demonstrated a decreased expression of Fas receptor or Fas ligand [22] in asthmatic subjects, while other studies have reported an increased expression of Fas receptor and Fas ligand in asthmatic subjects [23]. Several studies showed that SM induces apoptosis by both the intrinsic mitochondrial pathway and the extrinsic Fas-Fas ligand pathway [12, 15, 24–27]. In this study, there was significant increase in BAL fluid cells Fas and FasL levels in mild and moderate-to-severe patients compared to the control. FEV1/FVC ratio which is more sensitive for air way obstruction was significantly higher in patients with mild disorder compared with moderate-to-severe respiratory disease. It indicates that the patients with moderate-to-severe symptoms are more prone to airway obstruction. Our findings are concurrent with previously studies that reported increased Fas and FasL levels in BAL fluid of the patients with BO and acute respiratory distress syndrome compared with their corresponding control group. It has been suggested that it might play important role in pathology of respiratory dysfunction [28–30]. Rosenthal et al. [24] have shown that SM exposure destabilizes intracellular p53 leading to enhanced Fas receptor and FasL expression in human keratinocytes at vesicant SM concentrations. Ray et al. [31], also reported that acute exposure to various mustard concentrations would induces apoptotic pathway in normal human bronchial epithelial cells and small airway epithelial cells via caspase-mediated pathway in vitro. A recent study reported that soluble FasL was increased in serum of SM-exposed patients [32].

Caspases are protease enzymes that play important role in the apoptosis process. Both extrinsic and intrinsic apoptotic pathways activate caspase-3 which is the executioner caspase responsible for the end effects in the apoptosis process [12, 33, 34]. Previous studies showed that caspase activity is involved in the cell death pathway that is induced following exposure to mild concentrations of SM [11, 15, 24, 31, 35]. In present study, there was no difference in BAL fluid cells caspase-3 activity among patient and control groups. Since caspase-3 is considered to be one of the main effector caspases in apoptotic cell death, it could be concluded that apoptosis was impaired in BAL fluid cells of patient population. Caspase-3 is normally present in the cells in an inactive zymogens form and requires proteolytic processing before they become active. Cleavage is affected by the context of large multiprotein complexes known as DISC (death-inducing signaling complex) and the apoptosome, the formation of which requires several adaptor proteins [33, 34]. It could be assumed that SM exposure might affect apoptosis process by affecting a variety of factors. A second type of regulatory proteins that might be affected in SM-exposed patients could be FLICE inhibitory protein (FLIP). FLIP is able to block early events in Fas and TNF-receptor-like apoptosis-inducing ligand (TRAIL)/TNF family death receptor signaling by precluding caspase-8 recruitment to the DISC. FLIP is a caspase-8-like protein that interferes with caspase-8 binding to the DISC, thus preventing caspase-8 oligomerization and autoactivation [36, 37]. Finally, SM exposure might activate the inhibitor of apoptosis proteins (IAPs), which constitutes a family of evolutionarily conserved apoptotic suppressors. Members of the IAP-family proteins, such as X-linked inhibitor of apoptosis protein (XIAP), have been shown to bind to and inhibit activated caspase-3, -7, and -9 [38].

Therefore, it could be concluded that in SM-exposed patients one of these inhibitory factors might be activated and prevented, the Fas-FasL-induced apoptosis. Patients experienced inhalation injury have developed BO, which was confirmed with pathology and high-resolution CT studies [39, 40]. Besides our clinical data, pathologic finding showed that there was no differences between the low- and high-dose SM exposure after 20 years of initial SM exposure, suggesting that SM effects are not dependant on exposure severity [4]. Lung disease in this group of patients showed a slowly progressive nature. Long-term medical followup after exposure revealed that approximately 75%–80% of our patients experienced mild respiratory impairment and only 20% of them developed severe disease [41]. This finding was conflicting to posttransplant BO which is marked by progressive obstructive lung disease leading to a progressive decline in FEV1 caused by an inflammatory process of the airways identical with chronic allograft rejection [42]. It seems that the major difference is due to the etiology of lung injury, in which over 20 years after exposure most of our patients have shown that their disease has slowly progressive diseasenature, but not experienced without any cure even with different medications. It seems that optimal apoptotic pathway cascades (efferocytosis) are not triggered efficiently. Most in vitro and in vivo studies have planned to investigate cellular damage after a short time of injury, which represents the acute effect and cellular changes [11, 15, 24, 31, 35]. 

Phosphatidylserine is normally exposed on inner side of plasma membranes, and it becomes exposed on outer membrane surface of the cells undergoing apoptosis. Therefore, annexin V-FITC is able to detect the surface changes in membrane surface that occur early during apoptosis [43]. In this study, further analysis of BAL fluid cells with Annexin V-FITC versus propidium iodide confirmed that the majority of cells became necrotic and only a small portion became apoptosis. During apoptosis, the cell shrinks, while producing apoptotic bodies which are taken up by neighboring cells by phagocytosis. Very little cytoplasmtic material is released into the intercellular space and no or limited inflammation is caused in neighboring tissues. In contrast, during necrosis, the cellular contents are released uncontrolled into the cell's environment which results in damage of surrounding cells and a strong inflammatory response in the corresponding tissue [20, 21]. Based on previous reports, lung diseases are related to inflammatory processes that generate increased reactive oxygen species that correlate with disease severity [44–46]. Additionally, SM upregulates many inflammatory mediators including interleukin- (IL-) 1alpha, IL-1beta, IL-6, IL-8, tumor necrosis factor-alpha (TNF-alpha) and others [47–49]. The main findings of lavage fluid of sulfur mustard-injured patients are inflammation, tissue damage, and neutrophil excess [46]. The neutrophils were shown to produce several cytokines such as tumor necrosis factor and transforming growth factor-*β*, as an inflammatory factor [50], and express Fas ligand [32, 50]. A study shows that transforming growth factor-*β* plays a pivotal role in bronchiolitis obliterans pathogenesis [51]. A recent study shows that the level of transforming growth factor-*β* is significantly increased in lavage fluid of patients with sulfur mustard-exposure, compared with veterans not exposed to sulfur mustard [52]. A 6-month treatment with gamma interferon, an anti-inflammatory effect via down-regulation transforming growth factor-*β* gene expression, is associated with an improvement in the lung function in sulfur mustard exposed patients with bronchiolitis [53]. However, recent studies showed that the serum levels of inflammatory cytokines such as IL-8, IL-6 [54], IL-1alpha, IL-1beta, and TNF-alpha, except for IL-1Ra and MMP-9 [55], decreased in SM-exposed subjects. More local studies on the lung are needed to clarify the exact role of this cytokines and inflammation.

## 5. Conclusion

Our study showed that SM exposure inhibited Fas-FasL-induced apoptosis in BAL fluid cells of exposed patients. This inhibition might change PMN, granulocyte and lymphocyte turnover affect cytokine, chemokine, and protein secretion from these cells, and potentiate initiation of lung injury in SM-exposed patients. In future studies, exact mechanism of this inhibition should be addressed for probable therapeutic application. In addition, nonapoptotic importance of Fas-FasL-like macrophage and PMN activation could be investigated.

## Figures and Tables

**Table 1:** Characteristics of the study populations.

	Control	Mild	Moderate-severe	*P* value
	(*n* = 6)	(*n* = 10)	(*n* = 10)	
Sex	Male	Male	Male	—
Age	38.7 ± 6.1	39.5 ± 5.4	43.5 ± 5.3	.12
Height	172.2 ± 3.7	174.5 ± 6.3	168.3 ± 4.5	.42
Weight	74.5 ± 9.8	74.3 ± 10.8	75.4 ± 10.5	.6

Values are shown as mean ± SD.

**Table 2:** The pulmonary function indices of the study populations.

	Control	Mild	Moderate to severe
	(*n* = 6)	(*n* = 10)	(*n* = 10)
RV (% predicted)	105.9 ± 9.6	130.2 ± 27.7	160.3 ± 62.5
FVC (% predicted)	88.2 ± 5.9	69.7 ± 18.1*	66.1 ± 13.2*
FEV1 (% predicted)	90.7 ± 6.5	75.3 ± 16.8	58.2 ± 17.6**
FEV1/FVC (%)	88 ± 5.8	87.2 ± 9.3^#^	65.4 ± 8.7***

Values are shown as mean ± SD. *P < .05, **P < .01, and ***P < .001 versus control. ^#^ *P* < .001 versus moderate-severe group.

**Table 3:** Differential percentage counts of BAL fluid cells.

	Control	Mild	Moderate to severe
	(*n* = 6)	(*n* = 10)	(*n* = 10)
PMN (%)	10.6 ± 1.7	3.8 ± 1.7	18.6 ± 11.3
Lymph (%)	2.5 ± 0.5	2.3 ± 0.8	3.4 ± 1.5
Macrophage (%)	85 ± 21	93.8 ± 2.3	68 ± 21

Values are shown as means ± SD.
